# HIF1α-SP1 interaction disrupts the circ-0001875/miR-31-5p/SP1 regulatory loop under a hypoxic microenvironment and promotes non-small cell lung cancer progression

**DOI:** 10.1186/s13046-022-02336-y

**Published:** 2022-04-27

**Authors:** Dong Wu, Tingting Chen, Xuanna Zhao, Dan Huang, Jiawei Huang, Yujie Huang, Qiu Huang, Zhu Liang, Chunyuan Chen, Min Chen, Dongming Li, Bin Wu, Lixia Li

**Affiliations:** 1 Department of Respiratory and Critical Care Medicine, Affiliated Hospital of Guangdong Medical University, Zhanjiang, China; 2 Department of Cardiothoracic Surgery, Affiliated Hospital of Guangdong Medical University, Zhanjiang, 524000 China; 3 Cancer Hospital, Affiliated Hospital of Guangdong Medical University, Zhanjiang, 524000 China

**Keywords:** Non-small cell lung cancer, Has-circ-0001875, miR-31-5p, SP1, EMT, Hypoxia, Regulatory loop

## Abstract

**Background:**

Circular RNAs (circRNAs) play an important role in the progression of non-small cell lung cancer (NSCLC), especially under tumor hypoxia. However, the precise functions and underlying mechanisms of dysregulated circRNAs in NSCLC are largely unknown.

**Methods:**

High-throughput RNA sequencing was performed to identify significantly expressed circRNAs in NSCLC tissues. The functions of circ-0001875 in NSCLC cells were investigated in vitro and in vivo. The regulatory relationships of circ-0001875, miR-31-5p and SP1 were examined by dual luciferase reporter assays and rescue experiments. The signal pathway of epithelial-to-mesenchymal transition and the formation of filopodia were analyzed by western blot and immunofluorescence staining. The binding of SP1 to Alu elements was evaluated by RNA immunoprecipitation, and the HIF1α and SP1 interaction was detected by co-immunoprecipitation.

**Results:**

We identified the novel Has_circ_0001875 as a significantly upregulated circRNA in NSCLC tissues and cell lines. circ-0001875 promoted the proliferation and metastasis of NSCLC both in vitro and in vivo, and induced NSCLC cells to extend filopodia. Mechanistically, circ-0001875 sponged miR-31-5p to regulate SP1, influencing epithelial-to-mesenchymal transition via the TGFβ/Smad2 signal pathway. SP1 negatively regulated circ-0001875 formation through an AluSq-dependent feedback loop, which was disrupted by competitive binding of HIF1α to SP1 under hypoxia condition. The circ-0001875/miR-31-5p/SP1 axis was associated with the clinical features and prognosis of NSCLC patients.

**Conclusions:**

Our results revealed that the circ-0001875/miR-31-5p/SP1 axis and the complex regulatory loops influence NSCLC progression. These findings provide new insights into the regulation of circRNA formation under tumor hypoxia.

**Supplementary Information:**

The online version contains supplementary material available at 10.1186/s13046-022-02336-y.

## Introduction

Lung cancer, the most commonly diagnosed cancer, is the leading cause of cancer death worldwide in both sexes combined [1]. Approximately 85% of lung cancers are non-small cell lung cancer (NSCLC) [2]. NSCLC has a high mortality rate, which mostly results from metastasis in early stage [3]. Hypoxia in tumors occurs when new blood vessels can not keep up with tumor growth and leads to the elevation of hypoxia-inducible factor-1α (HIF1α), which plays a vital role in NSCLC cells epithelial-to-mesenchymal transition (EMT) [4, 5]. Malignant progression of tumor cells has been reported to be associated with EMT [6, 7], in which cells transition from epithelial cell characteristics to acquire more mesenchymal characteristics [8]. EMT also involves the concomitant formation of filopodia required for invasive growth[9], which allows cells to explore and move into the surrounding environment [10, 11]. The specificity protein 1 (SP1) is a well-known transcription factor involved in cancer proliferation and metastasis and has been shown to promote tumor cell EMT [12, 13].

Circular RNAs (circRNAs) are covalently closed continuous loops of RNA molecules originating from the back-splicing of pre-mRNA transcripts that show higher stability than linear mRNA [14]. circRNAs exert important biological functions by acting as microRNA (miRNA) sponges to regulate target genes [14, 15]. miRNAs are noncoding RNAs that bind to the 3’ untranslated region (UTR) of target mRNAs to regulate gene expression at the posttranscriptional level [16, 17]. Emerging evidence has shown that circRNAs and miRNAs exert vital roles in the progression of NSCLC [18, 19] as well as hypoxia [20, 21]. However, the underlying mechanisms by which dysregulated circRNAs influence NSCLC progression need to be explored.

Alu elements are a series of repeat sequences located in the intronic flanks of circularized exons. Alu elements facilitate RNA circularization through base-pair, bringing the splice site sequences into close proximity to each other to form a loop [22]. Recent studies have shown that proteins bind Alu elements to prevent splice sites from being close enough, thus leading to the decrease of circRNA formation. For example, ADAR1 binds Alu elements to prevent circRNA formation and expression [23]. SP1 also binds with high affinity to Alu elements [24, 25], but whether it influences RNA circularization is unknown.

In this study, we explored the role and formation mechanism of dysregulated circRNAs in NSCLC. We identified circ-0001875 as an oncogenic circRNA that promotes proliferative and metastatic phenotypes of NSCLC cells in vitro and in vivo. We found that circ-0001875 functions as miR-31-5p sponge to regulate SP1 in its effects on EMT. Moreover, we identified a SP1-AluSq-dependent feedback loop that slowed down circ-0001875 circularization, and these effects were disrupted by the competitive binding of HIF1α to SP1 under hypoxia. circ-0001875, miR-31-5p and SP1 expression were strongly correlated with the prognosis of NSCLC patients. Collectively, our results strongly indicated that circ-0001875 may be a potential diagnostic, treatment and prognostic marker for NSCLC.

## Methods

### Cell culture and transfection

NSCLC cell lines (A549, PC9, H838, H1299, 95D), human normal lung epithelial cells (BEAS-2B), and 293A cells were purchased from Chinese Academy of Sciences (Shanghai, China). All cells were cultured in DMEM medium (Gibco, CA, USA) containing 10% fetal bovine serum (FBS) in a humidified incubator containing 5% CO2 at 37 °C. For hypoxia studies, the cells were cultured at 37 °C under 1% O_2_ and 5% CO_2_ in a humidified hypoxic chamber (Forma Scientific, Marietta, OH, USA).

Small interfering RNAs (siRNAs) targeting circ-0001875 and SP1, miRNAs mimics, inhibitors and corresponding negative controls were purchased from GenePharma (Shanghai, China). The full-length cDNA sequence of circ-0001875 and SP1 were used to construct overexpression plasmids by GenePharma. All siRNAs, miRNA mimics and inhibitors were transiently transfected into cells with Lipofectamine RNAiMAX (Invitrogen), and the overexpression plasmids were transfected with Lipofectamine 3000 (Invitrogen). The circ-0001875 overexpression (pLCDH-circ-0001875) and knockdown vector (sh-circ-0001875) and the control vector (GenePharma) were transfected into A549 and H1299 cells. Cells were selected with puromycin (Sigma-Aldrich, USA) for 2–3 weeks to establish stable cell lines. Sequences of all constructs used in this study are listed in Table S1.

### Tissue samples

We acquired 72 pairs NSCLC and matched tumor-adjacent tissues from patients who underwent surgery without preoperative chemotherapy or radiotherapy at Affiliated Hospital of Guangdong Medical University between 2017 and 2020. All tissues were stored in liquid nitrogen. This research was approved by the Ethics Committee of Affiliated Hospital of Guangdong Medical University. All patients signed informed consent forms before research was conducted.

### Animal models

Four-week-old female nude mice (BALB/c) were purchased from the medical experimental Animal Center of Guangdong province (Guangdong, China).

For the subcutaneous tumor model, the armpits of mice were subcutaneously injected with transfected cells (6 × 10^6^ cells/mice, *n* = 9/group for overexpression groups, *n* = 6/group for knockdown groups). In one experiment, mice were randomized into two groups: control shRNA (sh-NC) and circ-0001875 shRNA (sh-circ-0001875). In a second experiment, mice were randomized into two groups: control vector (pLCDH-vector) and circ-0001875 overexpression vector (pLCDH-circ-0001875). Tumor growth was monitored and measured with a vernier caliper every week. Tumor volume was calculated with the formula: V = (width^2^ × length)/2. At 4 weeks after tumor formation, mice were euthanized and subcutaneous tumors were harvested for HE and IHC staining.

For metastasis studies, transfected cells were injected into the tail vein of mice (10 × 10^6^ cells/mice, *n* = 9/group for overexpression groups, *n* = 6/group for knockdown groups). After 6 weeks, lungs and livers were dissected, and all samples were weighed.

Animal experiments were approved by the animal ethics committee of Guangdong Medical University and were fed in the SPF grade of the Laboratory Animal Center of Guangdong Medical University.

#### RNA-seq

Total RNA was isolated from three pairs of NSCLC tissues and adjacent normal tissues by TRIzol Reagent (Invitrogen, CA, USA). The RNA integrity was assessed by Agilent 2100, samples with an RNA integrity number > 7.0 were used in experiments. Ribosomal RNA was removed from samples using a RiboMinus Eukaryote kit (Qiagen, Valencia, CA, USA). The RNA-seq library was deep sequenced using an Illumina HiSeq 2000 instrument (Illumina, San Diego, CA, USA) to harvest paired-end reads, and Edger software (v3.16.5) was used to identify the differentially expressed circRNAs.

#### RNA extraction and quantitative real-time polymerase chain reaction (RT-qPCR)

Total RNA was extracted from tissues and cells by Trizol reagent (Invitrogen) in accordance with the manufacturer’s instructions. cDNA was synthesized using Evo M-MLV RT Premix (AG, Hunan, China) and RT-qPCR was performed on an ABI7500 (Applied Biosystems, Foster City, CA, USA) or LightCycler 480 (Roche Applied Biosystems). U6 and β-actin mRNA were used as internal loading controls for miRNA and mRNA, respectively. Primers (Sango Biotech, Shanghai, China) used in the study are listed in Table S2. Each sample was replicated at least three times and data were analyzed by the 2-ΔΔCT statistical method.

#### RNase R treatment and actinomycin D (ActD) treatment

For RNase R treatment, total RNA extracted from cells (2 μg) was incubated at 37 °C for 15 min with 3U/μg RNase R (Epicentre Technologies Corporation, Madison, WI, USA). For ActD treatment, cells were cultured with 2 μg/mL ActD (Beyotime, Shanghai, China) for specific times.

#### Cell Counting Kit-8 (CCK8) assay and cloning formation assay

For CCK-8 assay (Beyotime), transfected cells were inoculated into 96-well plates at 2000 cells in 100 μl medium per well. At 0, 24, 48, 72 and 96 h after inoculation, 10 μl of CCK-8 reagent was added and cells were incubated at 37 °C for 1 h. Cell viability was detected at absorbance at 450 nm.

For cloning formation assay, transfected cells (500 cells/well) were inoculated into 6-well plates and cultured at 37 °C for 2 weeks. Cells were fixed in methanol for 30 min and then cells were stained with 0.1% crystal violet. The cells were imaged and counted.

#### Wound healing assay

Cells were inoculated into 6-well plates and transfected. Scratch wounds were generated in the cell monolayer by 1-ml pipette tip after cell density reached approximately 90%. Floating cells were removed by washing with 1 × PBS, and cells were incubated in serum-free medium. Wound healing was photographed at 0, 24 and 48 h using inversion microscopy. Cell migration was analyzed by ImageJ.

#### Migration and invasion assays

Approximately 3 × 10^5^ transfected cells in 200 μl serum-free medium were inoculated into the upper chamber of a Transwell chamber (BD Biosciences, Franklin Lakes, NJ, USA) with or without Matrigel (BD Biosciences) for invasion and migration assays, respectively. The lower chambers were filled with 750 μL medium containing 20% FBS. After incubation of culture plates for 24 h, cells were fixed with methanol and stained with 0.5% crystal violet (Beyotime). Migrated and invaded cells were imaged under a microscope.

#### Western blot

Transfected cells were lysed in RIPA (Beyotime) containing PMSF and protein concentrations were quantified with BCA reagent (Beyotime), Equal amounts of protein were electrophoresed on 10% SDS-PAGE gels and transferred onto PVDF membranes (Millipore, Billerica, MA, USA). After blocking the membranes with 5% defatted milk at room temperature for 1 h, the membranes were incubated with primary antibodies at 4 °C on a shaker overnight. The membranes were then incubated with indicated HRP-labeled secondary antibodies for 1 h. Protein signals were detected by BeyoECL star (Beyotime). All antibodies used in this study are listed in Table S3.

#### Fluorescence in situ hybridization (FISH) and immunofluorescence staining

Cells were fixed with 4% paraformaldehyde and permeabilized in PBS with 0.5% Triton X-100 for 30 min in a confocal dish. For FISH, cells were incubated with FITC-labeled circ-0001875 probe (GenePharma) at 37 °C overnight. To detect filopodia formation, cells were incubated with Phalloidin (YEASEN, Shanghai, China) for 90 min at 37 °C. Anti-fade mounting medium was added with DAPI staining, and signals were imaged by Olympus Laser confocal microscopy (Olympus Corporation, Tokyo, Japan).

#### RNA-binding protein immunoprecipitation (RIP) assay

RIP assays were performed using the Magna RIP RNA-Binding Protein Immunoprecipitation Kit (Millipore). Approximately 2 × 10^7^ cells were lysed in RIP lysis buffer containing protease and RNase inhibitors. Cell lysates were then incubated with IgG or anti-SP1 antibody conjugated magnetic beads (Millipore) with rotation at 4 °C overnight. The next day, the RNA/bead complexes were washed with RIP wash buffer and resuspended in Proteinase K buffer to purify immunoprecipitated RNA. RNA was reverse transcribed to cDNA and analyzed by RT-qPCR.

#### Co-immunoprecipitation (CoIP) assay

Transfected cells were lysed with RIPA buffer (Beyotime) and incubated with normal IgG, anti-SP1, or anti-HIF1α antibodies on a rotator at 4 °C overnight. Antibody-protein complexes were captured by incubation with Dynabeads-protein G beads (Beyotime) for 2 h. The complexes were then analyzed by western blot analysis.

#### Dual luciferase reporter assay

Luciferase reporter plasmids (wild-type and mutant-type) of circ-0001875 and SP1 were synthesized by GenePharma. Cells were inoculated into 24-well plates. When the cell density reached approximately 60%, luciferase plasmids, the Renilla control plasmid and miRNA mimics were transfected. Firefly and Renilla luciferase levels were detected using the Dual Luciferase Assay System (Promega).

#### Immunohistochemistry (IHC)

Tumor tissues from nude mice were cut into 4 μm slices for slide preparation, the slides were deparaffinized with xylene and rehydrated with ethanol. After incubation with 3% hydrogen peroxide to block endogenous peroxidase activity, the sections were soaked in sodium citrate buffer to recover antigen and 5% BSA was added to block nonspecific binding sites. The sections then incubated with anti-Ki67 and anti-SP1 primary antibodies at 4 °C overnight, followed by incubation with secondary antibodies. Images were photographed by a microscope (Olympus Corporation).

#### Bioinformatics analysis

Circ-0001875 sequence data were obtained from circBase. The target miRNAs of circ-0001875 were predicted by circular RNA interactome (https://circinteractome.nia.nih.gov). Target genes were predicted by TargetScan (https://www.targetscan.org/), miRbase (https://www.mirbase.org/), and miRDB (https://www.mirdb.org/).

#### Statistical analysis

GraphPad Prism 8.0 and SPSS 23.0 software were used for statistical analysis. Differences between data of two groups were analyzed by Student’s t-test, while differences between multiple groups were calculated by ANOVA. Survival curves were determined using the Kaplan–Meier method. Correlations between circ-0001875, miR-31-5p and SP1 were analyzed by Pearson’s correlation test. Differences were considered statistically significant at *P*-value < 0.05.

## Results

### Circ_0001875 is upregulated in NSCLC cells and tissues

**Fig. 1:**
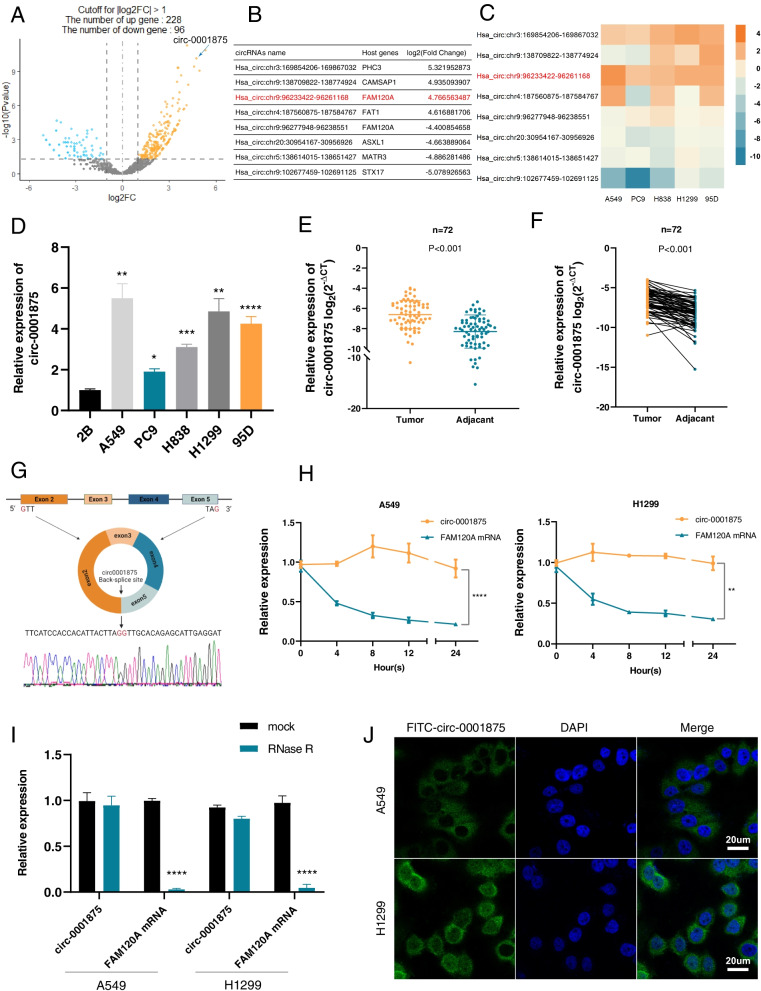
Expression and characterization of circ-0001875 in NSCLC cells and tissues. **a** Volcano plots showing the differentially expressed circRNAs in NSCLC tissue relative to matched normal tissue. **b** The top eight dysregulated circRNAs are listed. **c** RT-qPCR analysis of the eight most dysregulated circRNAs in A549, PC9, H838, H1299 and 95D cells compared with expression in BEAS-2B cells. **d** Relative expression levels of circ-0001875 in BEAS-2B cells and NSCLC cell lines were determined by RT-qPCR. **e–f** Relative expression of circ-0001875 in NSCLC tissues and adjacent nontumor tissues was detected by RT-qPCR (*n* = 72). **g** A schematic of the genomic location and back splicing of circ-0001875 with the splicing site validated by Sanger sequencing. **h–i** Circ-0001875 and *FAM120A* mRNA expression were detected in NSCLC cells after Actinomycin D and RNase R treatment. **j** FISH assay showed that circ-0001875 (FITC-labeled probe) was abundant in the cytoplasm, DAPI was used to stain cell nuclei. Images are shown at 600 × magnification. Scale bar = 20 μm. Data are shown as the means ± SD. *p < 0.05, **p < 0.01, ***p < 0.001

To explore the circRNAs that play a vital role of NSCLC, we performed RNA-seq on three pairs of NSCLC and matched normal lung tissues. The results identified 5976, 6547 and 8459 circRNAs in the three NSCLC samples and 6254, 4821 and 5419 circRNAs in the matched normal samples. Using the criteria of log2(fold-change) absolute value > 1 and *P*-value < 0.05, we identified 324 differentially expressed circRNAs, including 228 upregulated and 96 downregulated circRNAs (Fig. 1a). The top eight dysregulated circRNAs based on their fold-change were selected for further analysis (Fig. 1b). To rectify the “Type I” RNA-seq error, a heat map was used to visualize the variation in the expression of these circRNAs in five NSCLC cell lines compared with expression in the normal alveolar epithelial cell line BEAS-2B (Fig. 1c). circ-0001875 (chr9:96,233,422–96,261,168) was a significantly upregulated circRNA in both RNA-seq and NSCLC cell lines (Fig. 1d). A549 and H1299 cells showed relatively high circ-0001875 expression and were selected for subsequent studies. We further detected higher circ-0001875 expression in 72 NSCLC samples compared with expression in paired adjacent normal tissue samples using RT-qPCR, and these results were consistent with the RNA-seq data (Fig. 1e–f). The circBase (http://www.circbase.org/) annotation result showed that circ-0001875 is back-spliced from exon 2 to 5 of the *FAM120A* gene, with a length of 556 bp. The back-spliced junction of circ-0001875 was amplified using divergent primers and confirmed by Sanger sequencing (Fig. 1g)  [26]. To confirm circ-0001875 has the characteristic stability of circRNAs, divergent and convergent primers were designed to amplify the circular and linear form, circ-0001875 and *FAM120A* mRNA, respectively. Actinomycin D and RNase R assay indicated that circ-0001875 was more stable than the linear *FAM120A* mRNA (Fig. 1h–i). FISH assay results showed that circ-0001875 was primarily located in the cytoplasm (Fig. 1j). Together, these data demonstrated that circ-0001875 is an upregulated and highly stable circRNA that is located in the cytoplasm of NSCLC cells.

### Circ_0001875 promotes the proliferation and metastasis of NSCLC cells both in vitro and in vivo

**Fig. 2:**
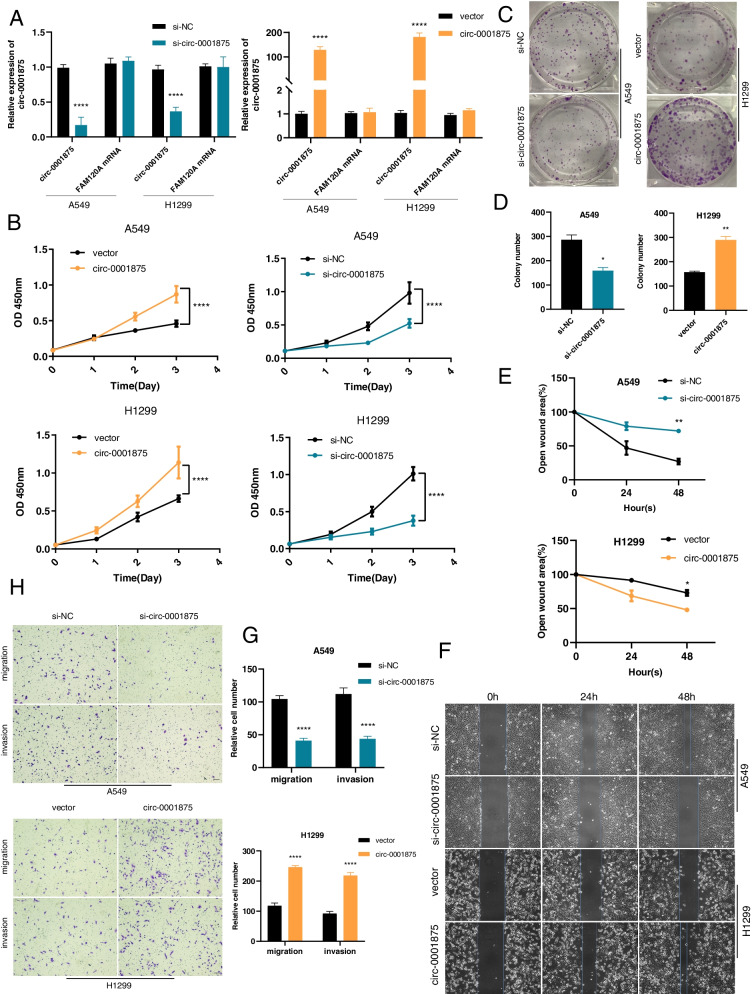
Circ_0001875 promotes the proliferation, migration and invasion of NSCLC cells in vitro. **a** Relative expression of circ-0001875 and *FAM120A* mRNA in A549 and H1299 cells after transfection with circ-0001875 siRNA and overexpression vector (si-circ-0001875 and circ-0001875) and the controls (si-NC and vector). **b–d** CCK8 and colony formation assays were performed to evaluate cell proliferation in the indicated cell lines. **e–f** Wound healing assay was used to assess the migration ability in A549 and H1299 cells transfected as indicated. **g–h** The migration and invasion abilities of cells transfected as indicated were detected by Transwell assays. Images are shown at 100 × magnification. Scale bar = 100 μm. Data are shown as the means ± SD. *p < 0.05, **p < 0.01, ***p < 0.001

To evaluate the effects of circ-0001875 on the biological behavior of NSCLC cells, we performed in vitro functional assays and in vivo animal experiments. First, we constructed two siRNAs that target the back splice sites of circ-0001875 and chose the most effective siRNA for further studies (Fig. S1a). A circ-0001875-overexpressing plasmid was used to overexpress circ-0001875 in NSCLC cells. The silencing and upregulation of circ-0001875 were confirmed in cells transfected with these constructs, with no influence on *FAM120A* expression (Fig. 2a). We next used CCK8 and colony formation assays to evaluate the effects of circ-0001875 on cell proliferation in A549 and H1299 cells. The results indicated that knockdown of circ-0001875 inhibited cell proliferation, while circ-0001875 overexpression significantly promoted cell growth (Fig. 2b–d and Fig. S1b–c). Furthermore, Transwell and wound healing assay demonstrated that cell migration and invasion activities were inhibited upon circ-0001875 silencing, while upregulation of circ_0001875 led to the opposite effects (Fig. 2e–h and Fig. S1d–g). These in vitro results indicated that circ-0001875 functions as an oncogene in NSCLC cells.

**Fig. 3:**
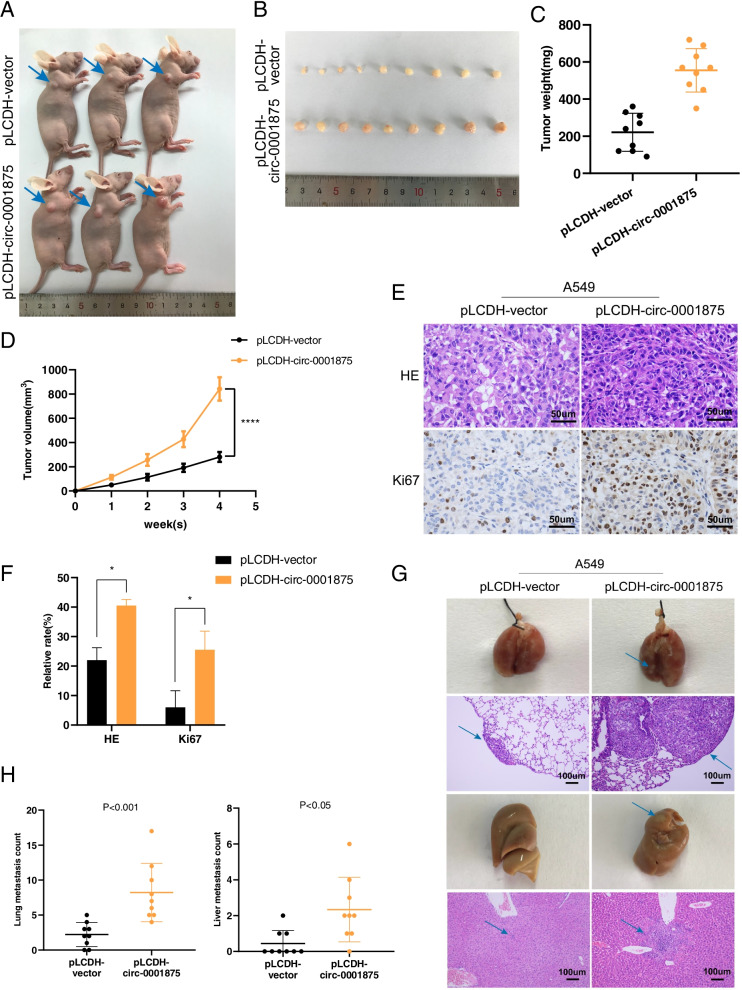
Circ_0001875 promotes the tumorigenesis and metastasis of NSCLC cells in vivo. **a** Representative images of a subcutaneous tumor nude mouse model. Hypodermic injection of A549 cells stably transfected with lentivirus expressing the circ-0001875 overexpression construct (pLCDH-circ-0001875) or control vector (pLCDH-vector) was performed in nude mice. **b** Images of subcutaneous xenograft tumors (*n* = 9 for each group). **c** Tumor weight of the two groups. **d** Tumor volume was measured every week. **e–f** HE and Ki67 IHC staining of xenograft tumors, images are shown at 400 × magnification. Scale bar = 50 μm. **g** Representative images of HE staining in tail vein–injected mouse models (*n* = 9 for each group), images are shown at 100 × magnification. Scale bar = 100 μm. **h** The numbers of lung and liver metastatic tumors were counted. Data are shown as the means ± SD. *p < 0.05, **p < 0.01, ***p < 0.001

To explore the effects of circ-0001875 on the proliferation and migration abilities of NSCLC cells in vivo, we constructed a subcutaneous nude mouse tumor model using A549 and H1299 cells stably transfected with pLCDH-circ-0001875 or pLCDH-vector and sh-circ-0001875 or sh-NC. Overexpression of circ-0001875 promoted tumor growth in the subcutaneous tumor model compared with that in the vector control group (Fig. 3a–b), and higher tumor weights and volumes were detected in the overexpression group (Fig. 3c–d). IHC staining showed that Ki67 expression was increased in tumors with upregulated circ-0001875 (Fig. 3e–f). Opposite results were observed in the circ-0001875 silenced group (Fig. S2a–e).

To further explore the function of circ-0001875 in cell migration in vivo, transfected cells were injected into the tail veins of nude mice. The results revealed significantly increased numbers of metastatic nodules in the lungs and livers in the circ-0001875 overexpression group (Fig. 3g–h) and decreased numbers in the circ-0001875 knockdown group (Fig. S2f–g). Together, these results demonstrated that circ-0001875 promotes NSCLC proliferation and metastasis both in vitro and in vivo.

### Circ_0001875 functions as a sponge for miR-31-5p

**Fig. 4:**
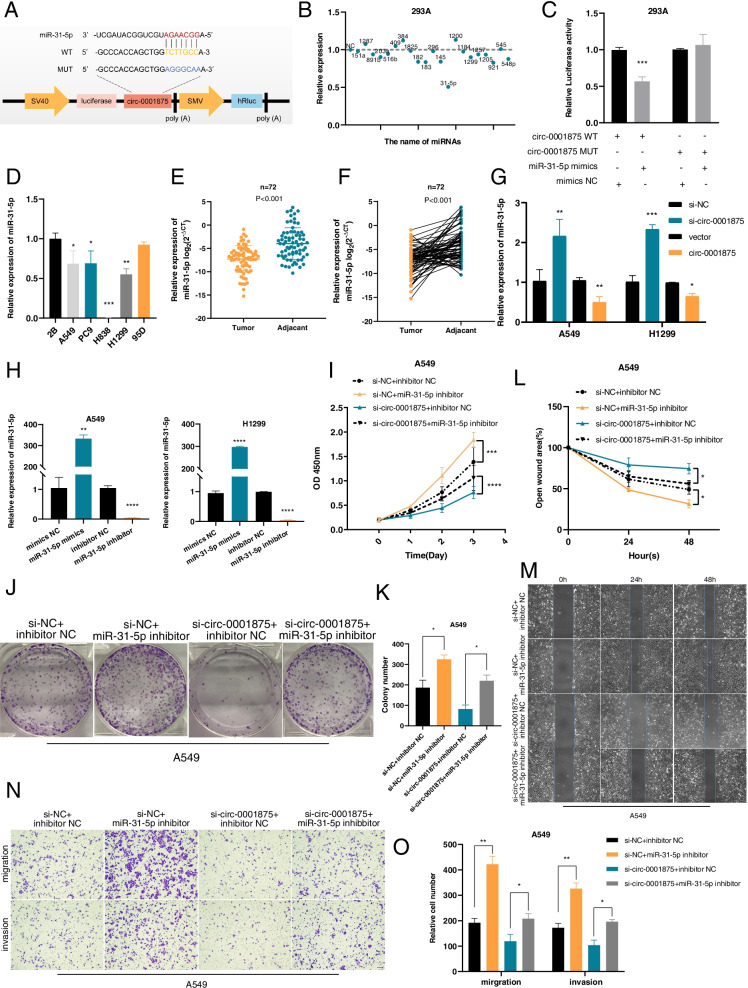
Circ_0001875 acts as a sponge for miR-31-5p. **a** Schematic of the luciferase reporter plasmids of circ-0001875 wild-type (circ-0001875 WT) and mutant-type (circ-0001875 MUT). **b** Relative luciferase activities of the circ-0001875 WT in 293A cells transfected with 21 candidate miRNAs mimics or mimic NC. **c** Luciferase activities of the circ-0001875 WT and circ-0001875 MUT transfected with miR-31-5p mimics or mimic NC. **d** RT-qPCR of the relative expression levels of miR-31-5p in NSCLC various cell lines. **e–f** Relative expression of miR-31-5p in NSCLC tissues and adjacent nontumor tissues was detected by RT-qPCR (*n* = 72). **g** Relative expression of miR-31-5p in A549 and H1299 cells transfected as indicated as detected by RT-qPCR. **h** Relative expression of miR-31-5p in A549 and H1299 cells transfected with the miR-31-5p mimics and inhibitor was detected by RT-qPCR. **i–o** Rescue experiments. CCK8, colony formation, wound healing assay and Transwell assays were conducted in cells in four treatment groups (si-NC + inhibitor NC, si-NC + miR-31-5p inhibitor, si-circ-0001875 + inhibitor NC, si-circ-0001875 + miR-31-5p inhibitor). Images are shown at 100 × magnification. Scale bar = 100 μm. Data are shown as the means ± SD. *p < 0.05, **p < 0.01, ***p < 0.001

circRNAs exert significant functions by acting as miRNA sponges through complementary base pairing with the “seed” region of miRNAs [18, 27, 28]. Therefore, to examine whether circ-0001875 functions as a miRNA sponge, we predicted potential target miRNAs of circ-0001875 using a prediction database Circinteractome [29] and 21 candidate miRNAs were identified (Table S4). Subsequently, we performed a series of dual luciferase assays. We constructed a luciferase reporter plasmid containing wild-type circ-0001875 (circ-0001875 WT) (Fig. 4a). We co-transfected the luciferase reporter plasmic containing circ-0001875 WT and the 21 candidate miRNAs in 293A cells; the luciferase activity was reduced after circ-0001875 WT was co-transfected with miR-31-5p mimics (Fig. 4b and c). Therefore, we selected miR-31-5p for subsequent studies. We next constructed a mutant luciferase reporter plasmid (circ-0001875 MUT) based on the predicted binding sites with miR-31-5p (Fig. 4a), and observed no changes in luciferase activity with co-transfected miR-31-5p and the circ-0001875 MUT reporter (Fig. 4c). Additionally, miR-31-5p was expressed at low levels in NSCLC cell lines and tissues (Fig. 4d–f). circ-0001875 knockdown increased the expression of miR-31-5p, while upregulated circ-0001875 reduced the expression levels of miR-31-5p (Fig. 4g). These results indicated that circ-0001875 suppressed miR-31-5p expression and functions as a sponge to bind miR-31-5p at the “seed” region.

To explore whether circ-0001875 exerts its function by sponging miR-31-5p, we used miR-31-5p mimics and inhibitor to modulate miR-31-5p expression in A549 and H1299 cells, as confirmed by RT-qPCR (Fig. 4h). Rescue experiments showed that miR-31-5p inhibitor promoted proliferation, migration and invasion in A549 cells and partly attenuated si-circ-0001875-mediated inhibition of these biological functions as shown in CCK8, colony formation, wound healing assay and Transwell assays (Fig. 4i–o). Similar results were observed after co-transfection with miR-31-5p mimics and the circ-0001875 plasmid in H1299 cells (Fig. S3a–g). Together, these results demonstrated that miR-31-5p may function as a tumor suppressor and serves important functions downstream of circ-0001875.

### SP1 is a target gene of miR-31-5p and indirectly regulated by circ-0001875

**Fig. 5:**
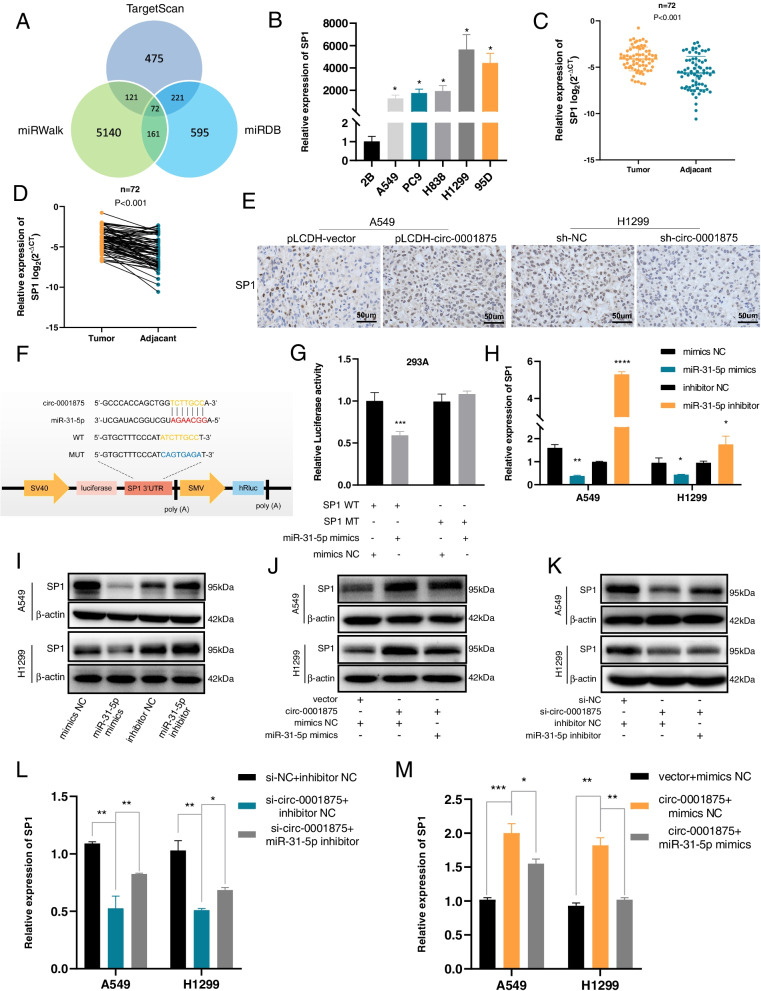
SP1 is a target gene of miR-31-5p and indirectly regulated by circ-0001875. **a** Venn diagram showing 72 genes that are putative miR-31-5p targets predicted by three databases (Targetscan, miRDB and miRWalk). **b** RT-qPCR of the expression of SP1 mRNA in NSCLC cells. **c–d** Relative expression of SP1 in NSCLC tumor and adjacent tissues (*n* = 72). **e** IHC staining of SP1 expression in xenograft tumors in the four groups (circ-0001875 overexpression vector (pLCDH-circ-0001875), control vector (pLCDH-vector), circ-0001875 shRNA (sh-circ-0001875) and control shRNA (sh-NC)). The images are shown at 400 × magnification. Scale bar = 50 μm. **f** A schematic of the SP1 wild-type (SP1 WT) and mutant-type (SP1 MUT) luciferase reporter plasmids. **g** Relative luciferase activities of the SP1 WT and SP1 MUT reporters in 293A cells transfected with miR-31-5p mimics or mimic NC. **h–i** RT-qPCR and western blot of SP1 mRNA and protein in cells transfected with miR-31-5p mimics and inhibitor. **j–m** Relative expression of SP1 at both the mRNA and protein levels in the indicated groups (si-NC + inhibitor NC, si-circ-0001875 + inhibitor NC, si-circ-0001875 + miR-31-5p inhibitor and vector + mimics NC, circ-0001875 + mimics NC, circ-0001875 + miR-31-5p mimics). Data are shown as the means ± SD. *p < 0.05, **p < 0.01, ***p < 0.001

To further explore the potential mechanism associated with the circ-0001875-targeted miR-31-5p regulation axis, we performed a cross-analysis using three gene prediction databases (TargetScan, miRDB and miRWalk) and identified 72 potential overlapping genes that may be targeted by miR-31-5p (Fig. 5a) [30–32]. After review of the literature of the candidate genes, we found that SP1 was regulated by miR-31-5p to promote cancer proliferation, migration, and invasion [33] and is also involved in lung cancer proliferation and metastasis processes [13]. Therefore, we selected SP1 for further studies. Similar to the patterns of circ-0001875 expression in NSCLC cell lines and tissues, higher expression of SP1 was observed in the tumor samples compared with that in normal samples (Fig. 5b–d). IHC staining results for SP1 in the subcutaneous tumors from the mice described above further verified the consistency between circ-0001875 and SP1 expression (Fig. 5e). According to the prediction databases used above, SP1 and circ-0001875 share the same “seed” region of miR-31-5p. Therefore, we constructed a luciferase reporter plasmid of SP1 (SP1 WT) and a construct with the mutated “seed” region (SP1 MUT) (Fig. 5f). Co-transfection of miR-31-5p mimics in 293A cells resulted in significant downregulation of luciferase activity from the SP1 WT reporter but not the SP1 MUT reporter (Fig. 5g). RT-qPCR and western blot results showed that SP1 was negatively regulated by miR-31-5p, with reduced expression in cells transfected with miR-31-5p mimics, while the opposite trend was observed in cells transfected with the inhibitor (Fig. 5h–i). Moreover, SP1 mRNA and protein levels were positively affected by circ-0001875, and these effects were partly rescued by miR-31-5p mimics or inhibitor treatment (Fig. 5j–m). Together, these results demonstrated SP1 is a downstream target of miR-31-5p and is indirectly regulated by circ-0001875.

### Circ_0001875 exerts its effects on malignant phenotypes of NSCLC cells via the miR-31-5p/SP1 axis

**Fig. 6:**
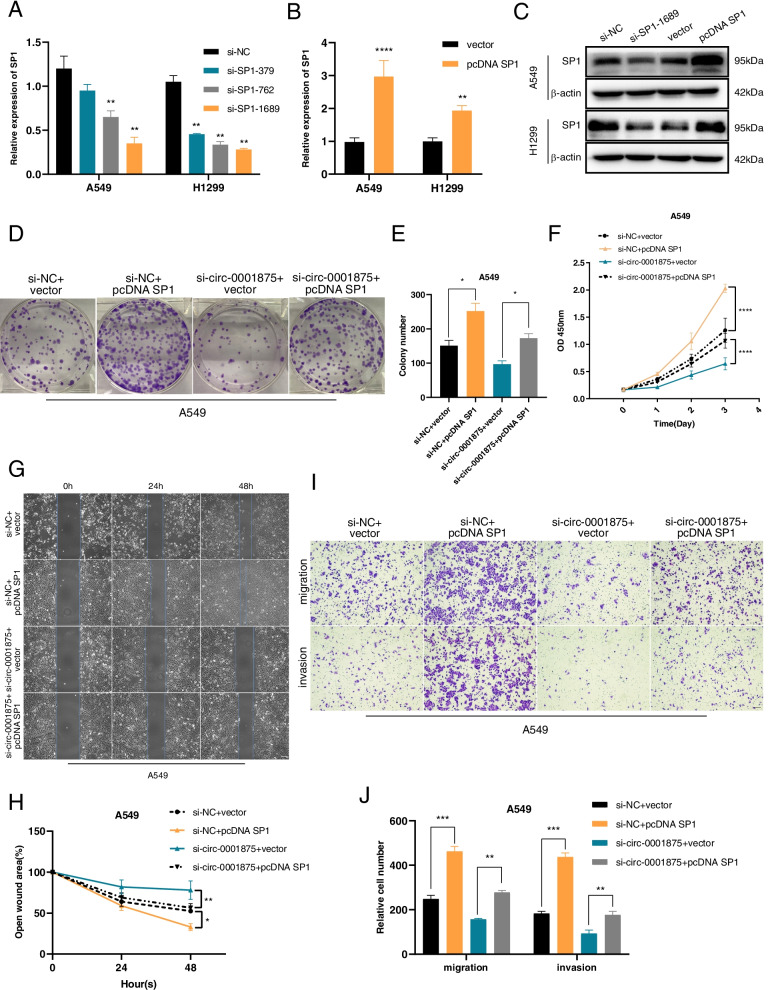
Circ_0001875 regulates malignant phenotypes in lung cancer cells via the miR-31-5p/SP1 axis. **a–c** Relative expression of SP1 mRNA and protein in cells transfected with three short hairpin RNAs (si-SP1) and an overexpression vector of SP1 (pcDNA SP1). **d–j** CCK8, colony formation, wound healing assay and Transwell assays in the four experimental groups shown (si-NC + vector, si-NC + pcDNA SP1, si-circ-0001875 + vector, si-circ-0001875 + pcDNA SP1). Images were obtained at 100 × magnification. Scale bar = 100 μm. Data are shown as the means ± SD. *p < 0.05, **p < 0.01, ***p < 0.001

To further evaluate whether SP1 functions downstream of miR-31-5p and circ-0001875 in NSCLC cells, we modulated SP1 levels using three siRNAs and an overexpression plasmid and confirmed changes in mRNA and protein levels in A549 and H1299 cells (Fig. 6a–c). CCK8, colony formation, wound healing and Transwell assays showed that SP1 overexpression promoted the proliferation, migration and invasion of A549 cells (Fig. 6d–j), while the opposite effect was observed by silencing SP1 in H1299 cells (Fig. S4a–g). Furthermore, the enhancement of malignant biological phenotypes in cells with augmented SP1 expression was reduced by silencing circ-0001875 (Fig. 6d–j). In contrast, the increased proliferation, migration and invasion capabilities of NSCLC cells caused by circ-0001875 overexpression were reduced by SP1 knockdown (Fig. S4a–g). Together, these results indicated that circ-0001875 exerts its oncogenic functions in NSCLC through the miR-31-5p/SP1 axis.

### Circ_0001875 via the miR-31-5p/SP1 axis promotes EMT through a TGFβ/Smad2 signal pathway

**Fig. 7:**
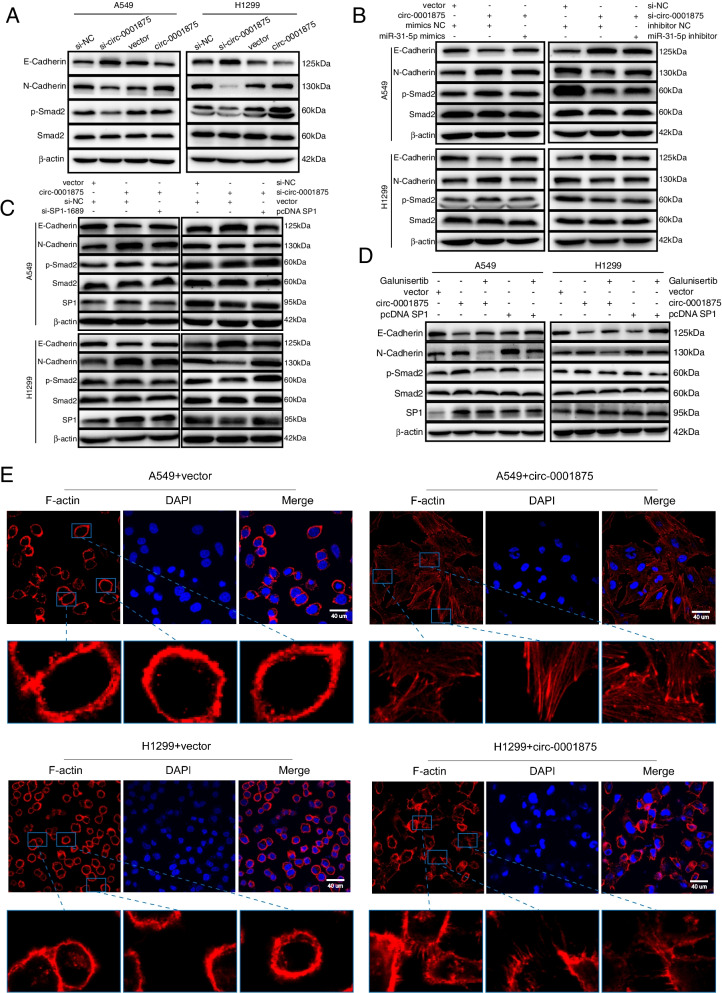
Circ_0001875 via the miR-31-5p/SP1 axis promotes EMT through a TGFβ/Smad2 signal pathway. **a** Western blot analysis of p-Smad2, Smad2 and EMT-associated molecules in cells with circ-0001875 knockdown and overexpression treatment. **b–c** Western blot analysis of p-Smad2, Smad2 and EMT-associated molecules in cells treated as indicated. **d** Western blot analysis of p-Smad2, Smad2, EMT-associated molecules and SP1 were analyzed in cells transfected with the indicated vectors and treated with TGFβ/Smad pathway inhibitor (Galunisertib). **e** Immunofluorescent staining of F-actin to detect filopodia in the circ-0001875 overexpression group and vector group. Images were obtained at 600 × magnification. Scale bar = 40 μm. Data are shown as the means ± SD. *p < 0.05, **p < 0.01, ***p < 0.001

EMT exerts a vital role in NSCLC metastasis [8]. In the EMT process, actin cytoskeleton reorganization results in filopodia formation, which is involved in EMT-driven cell invasion [9, 34]. miR-31-5p and SP1 has been reported to be associated with tumor EMT [35, 36]. Thus, we next explored whether the circ-0001875/miR-31-5p/SP1 regulation axis influenced EMT of NSCLC cells. We performed western blot analysis to evaluate the expression of EMT markers. Cells with knockdown of circ-0001875 showed increased levels of E-cadherin and reduced N-cadherin and p-Smad2 expression, while the opposite trends were observed by circ-0001875 overexpression (Fig. 7a), these effects were reversed by miR-31-5p inhibitor and mimics (Fig. 7b) or by SP1 overexpression and downregulation, respectively (Fig. 7c). Galunisertib is a potent and highly selective TGFβ/Smad pathway inhibitor [37]. circ-0001875 and SP1 overexpression reduced E-cadherin levels and increased N-cadherin and p-Smad2 expression, and these effects were reversed upon treatment with Galunisertib (Fig. 7d). Filopodia formation was observed in cells with circ-0001875 overexpression as detected by staining with F-actin (Fig. 7e). Together, these data further showed that the circ-0001875/miR-31-5p/SP1 axis mediated EMT of NSCLC cells via the TGFβ/Smad2 signal pathway and that circ-0001875 induced the formation of filopodia, which may be involved in EMT process.

### SP1 binds to AluSq elements forming a feedback loop to suppress circ-0001875 circularization

**Fig. 8:**
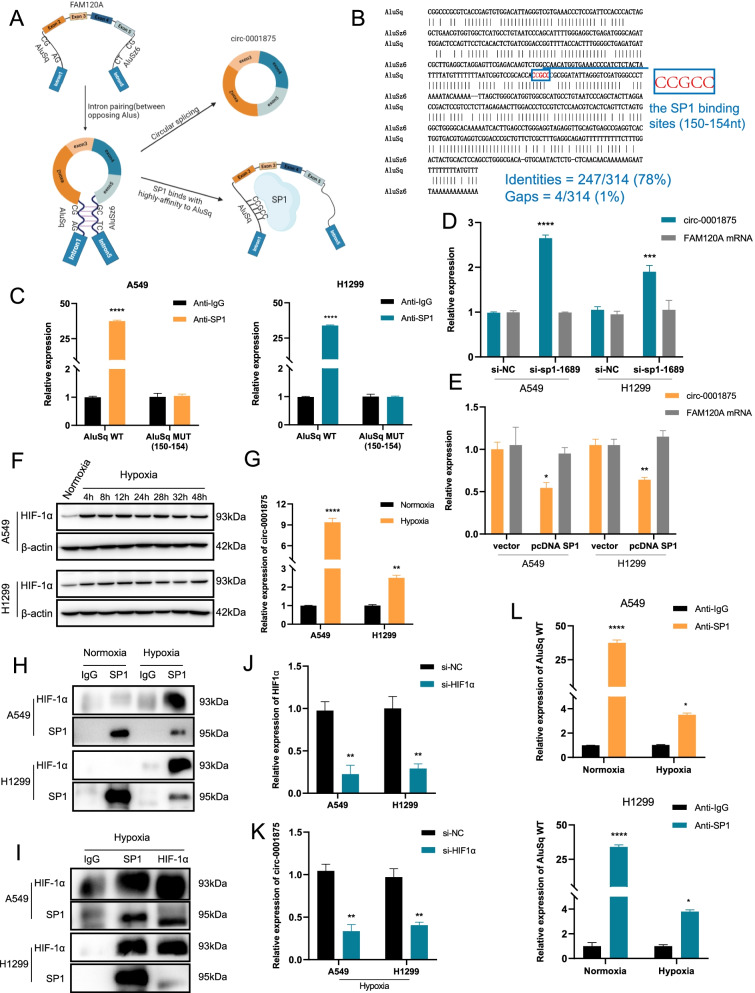
Hypoxia-induced HIF1α-SP1 interaction disrupts the circ-0001875/ miR-31-5p/SP1/AluSq regulatory loop. **a** Schematic diagram illustrates Alu-mediated RNA circularization and suppression when SP1 binds to AluSq element. **b** The sequences and base pairing condition of AluSq and AluSz6 and the SP1 binding sites on AluSq sequences are listed. **c** RIP assays using SP1 antibody were performed to examine interactions with the AluSq wild-type (AluSq WT) and AluSq 150–154 nt mutant-type (AluSq MUT (150–154)). **d–e** Relative expression of circ-0001875 and *FAM120A* mRNA in A549 and H1299 cells transfected with the indicated vectors or siRNAs was determined by RT-qPCR. **f** Western blot analysis of HIF1α expression in cells under hypoxia or normoxia. **g** circ-0001875 expression under hypoxia and normoxia analyzed by RT-qPCR. **h-i** CoIP analysis of the HIF1α-SP1 interaction in cells treated as indicated. **j** Relative expression of HIF1α in A549 and H1299 cells transfected with the short hairpin RNA of HIF1α (si-HIF1α) and determined by RT-qPCR. **k** Relative expression of circ-0001875 after transfected with si-HIF1α under hypoxia condition. **l** RIP assays using a SP1 antibody were performed to examine interactions with AluSq WT after NSCLC cells were treated with hypoxia. Data are shown as the means ± SD. *p < 0.05, **p < 0.01, ***p < 0.001

Alu elements located in the intronic flanks base-pair with one another, thereby bringing the splice sites into close proximity to each other and facilitating RNA circularization (Fig. 8a) [22]. However, this function can be disrupted when bound to ADAR1 or DHX9 [23, 38]. In addition, SP1 has shown to bind with high affinity to Alu elements [24, 25]. Based on these studies, we speculated that SP1 binds to Alu elements and may affect circ-0001875 circularization. To test our hypothesis, we performed a series of experiments. First, we found two Alu elements located in introns 1 and 5, flanking exons 2–5, named AluSq (range = chr9:96,230,149–96,230,464) and AluSz6 (range = chr9:96,261,559–96,261,869) (Fig. 8a), the sequences are listed in Fig. 8b. The upstream AluSq element is highly complementary to the downstream AluSz6 element (78% identity over 314nt) after base matching (Fig. 8b). The results suggested that the circ-0001875 circularization of exons 2–5 may be caused by the base complementary pairing of AluSq and AluSz6. We also identified SP1 binding sites to upstream AluSq (150–154 nt) (Fig. 8b). Therefore, we constructed a wild-type primer of AluSq containing the bases (AluSq WT) and a mutant primer (AluSq MUT (150–154)). We performed RIP assay and the results showed that AluSq WT expression was increased in SP1 immunoprecipitants, while AluSq MUT (150–154) was not (Fig. 8c), revealing that SP1 directly bound to AluSq on the 150–154 sites. Furthermore, RT-qPCR data showed that the silencing of SP1 significantly increased the expression of circ-0001875, while SP1 overexpression led to the decrease of circ-0001875, while linear *FAM120A* mRNA were meaningless changed at both (Fig. 8d–e). Collectively, these results suggesting SP1 binds upstream AluSq elements to prevent splice sites from being close enough to back splice and suppress circ-0001875 circularization (Fig. 8a). The circ-0001875/miR-31-5p/SP1 axis forms a feedback loop to regulate circ-0001875 expression through SP1-AluSq binding.

### Hypoxia-induced HIF1α-SP1 interaction disrupts the circ-0001875/miR-31-5p/SP1/AluSq feedback loop

Tumor growth leads to hypoxia [4]. To explore whether the circ-0001875/miR-31-5p/SP1/AluSq feedback loop is connected with hypoxia, we performed experiments in NSCLC cells under hypoxia. First, higher HIF1α expression under hypoxia condition compared with levels in normoxia condition, confirming the hypoxia model (Fig. 8f). While we observed high expression of circ-0001875 in NSCLC cells under normoxic culture (Fig. 1d), we observed significantly increased levels of circ-0001875 in cells treated with hypoxia for 24 h (Fig. 8g), suggesting that hypoxia promoted circ-0001875 circularization. Tumor hypoxia induces HIF1α expression to regulate gene expression [5] and the HIF1α-SP1 interaction also has been reported to associate with gene expression [39, 40]. We then explored whether the mechanism of hypoxia increased circ-0001875 expression involved the HIF1α-SP1 interaction. CoIP analysis in NSCLC cells after hypoxia for 24 h showed that HIF1α was immunoprecipitated with SP1 but not under normoxia condition (Fig. 8h). Overexpression of SP1 in cells under hypoxia condition and immunoprecipitation with anti-SP1 or anti-HIF1α antibodies confirmed the binding between HIF1α and SP1 (Fig. 8i). These results confirmed that SP1 and HIF1α interact during hypoxia. In addition, after modulating HIF1α levels using si-HIF1α in NSCLC cells (Fig. 8j), we found that the expression of circ-0001875 was reduced under hypoxia condition (Fig. 8k). Furthermore, RIP assay showed that binding between SP1 and AluSq was reduced in hypoxia than in normoxia (Fig. 8l). Collectively, these results suggested that HIF1α positively regulated circ-0001875 expression through competitive binding of SP1 with AluSq element under hypoxia condition.

### Circ_0001875/miR-31-5p/SP1 axis correlates with NSCLC prognosis

**Fig. 9:**
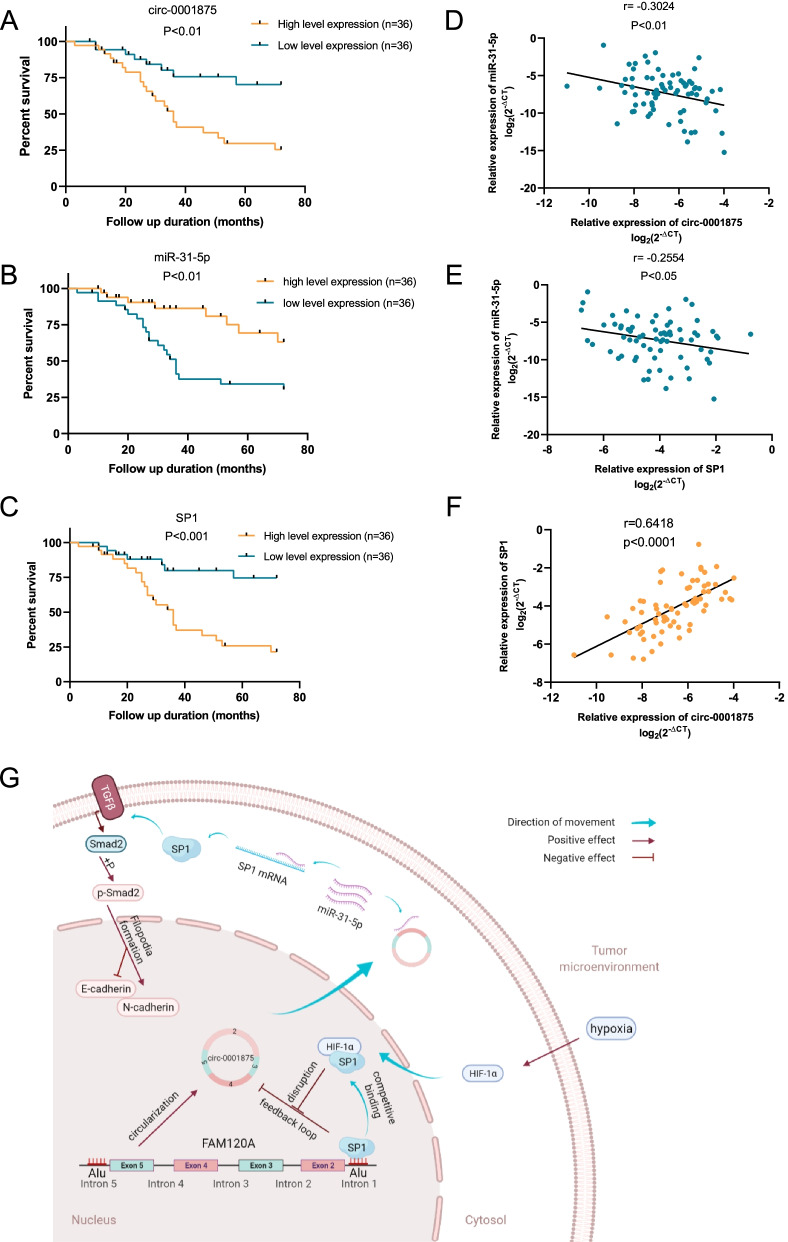
Circ_0001875/miR-31-5p/SP1 axis correlates with NSCLC prognosis and the schematic diagram illustrates the mechanism. **a–c** Kaplan–Meier survival curves showed the overall survival of NSCLC patients with low and high circ-0001875, miR-31-5p or SP1 expression (*n* = 72). **d–f** Correlation analysis between circ-0001875, miR-31-5p and SP1 expression analyzed by RT-qPCR in NSCLC tissues (*n* = 72). **g** Schematic diagram illustrates the mechanism by which the circ-0001875/miR-31-5p/SP1 and EMT axis promotes NSCLC proliferation and metastasis and negatively regulates by a SP1-AluSq-dependent feedback loop, but hypoxia-induced HIF1α-SP1 interaction disrupts the circ-0001875/miR-31-5p/SP1/AluSq feedback loop. The schematic diagram was elaborated by BioRender. Data are shown as the means ± SD. *p < 0.05, **p < 0.01, ***p < 0.001

We next evaluated the clinical significance of circ-0001875, miR-31-5p and SP1 in NSCLC patients. We used the median expression level as a cut-off value to categorize patients into high and low expression groups. Kaplan–Meier analysis showed that higher circ-0001875 expression, lower miR-31-5p expression and higher SP1 expression were each associated with poorer overall survival in NSCLC patients (Fig. 9a–c). The relationship between circ-0001875, miR-31-5p and SP1 expression and the clinical characteristics of the NSCLC patients are listed in Table 1, which showed that the smoking status and T stage^1^ correlated with high circ-0001875 and SP1 expression, respectively. Pearson correlation analysis indicated that circ-0001875 and SP1 expression in NSCLC tissues were negatively connected with miR-31-5p expression levels, while circ-0001875 was positively related with SP1 (Fig. 9d–f). Collectively, these results revealed the clinical significance of the circ-0001875/miR-31-5p/SP1 axis in NSCLC.

**Table 1:**
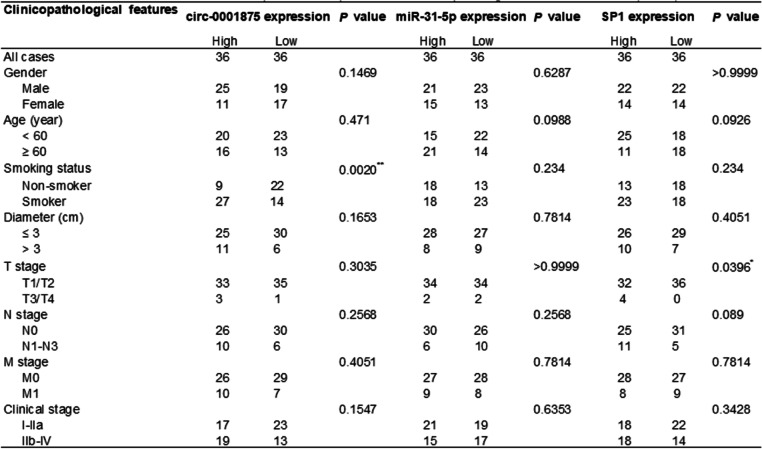
Association of circ-0001875, miR-31-5p and SP1 expression with clinicopathological features of NSCLC (*n* = 72)

Data are shown as the means ± SD. *p < 0.05, **p < 0.01

## Discussion

Accumulating evidence has demonstrated that dysregulated circRNAs exhibit critical functions in cancer progression [41]. In this study, we assessed differentially expressed circRNAs in three pairs of NSCLC tumor tissues and adjacent normal tissues by RNA sequencing and identified 324 differentially expressed circRNAs, including 228 upregulated and 96 downregulated circRNAs. Subsequent analysis identified circ-0001875 for further study of its contribution to NSCLC progression. circ-0001875 showed significantly high expression in NSCLC tissues and cell lines, and functional experiments showed that circ-0001875 promotes the proliferation, migration and invasion of NSCLC cells in vitro and in vivo, revealing an oncogenic role of circ-0001875 in NSCLC and highlighting the potential of circ-0001875 as a diagnostic and prognostic biomarker of NSCLC patients.

Many studies have demonstrated that circRNAs exert vital biological functions by acting as miRNA sponges. For example, cicEYA3 sponges miR-1294 to induce energy production in pancreatic ductal adenocarcinoma progression[42]. Guo. et al. showed that circ3823 contributes to the progression of colorectal cancer through competitively binding endogenous miR-30c-5p [43]. We observed that miR-31-5p was the target miRNA of the sponge function of circ-0001875 as demonstrated by expressions in NSCLC cell lines, tissues and luciferase activity assays. Further data illustrated the negative regulatory relationship between circ-0001875 and miR-31-5p. Rescue experiment results showed that miR-31-5p inhibitor rescued the inhibitory effect caused by circ-0001875 knockdown on proliferation, migration and invasion, and the promotion effect induced by circ-0001875 overexpression was interrupted by miR-31-5p mimics treatment. Our results demonstrated that circ-0001875 functions as an miR-31-5p sponge that is vital for the progression of NSCLC.

Zhao. et al. found that miR-31-5p targets SP1 to promote cell proliferation and migration in hepatocellular carcinoma [33]. In our study, SP1 was a candidate gene predicted by bioinformatic analysis. Further research was performed to explore whether circ-0001875 regulates miR-31-5p targeting SP1 to promote NSCLC cells malignant biological properties. We identified SP1 as a significantly upregulated gene in NSCLC tissues and cell lines, which was similar to the circ-0001875 expression pattern, and dual luciferase assay results verified that the SP1 is the target gene of miR-31-5p. Moreover, RT-qPCR and western blot results indicated that SP1 positively correlated with circ-0001875 expression and negatively correlated with miR-31-5p, when cells are co-transfected with circ-0001875 and miR-31-5p, SP1 expression can be rescued by miR-31-5p. Functional experiments showed that SP1 rescued the inhibitory effect induced by circ-0001875 silencing on proliferation, migration and invasion, while the opposite effect was observed in the SP1 overexpression group. Together, these results revealed that circ-0001875 promotes the progression and metastasis of NSCLC through the miR-31-5p/SP1 axis.

Cancer cells gain migratory and invasive properties in EMT, and EMT involves the formation of filopodia [9]. Western blot analysis revealed higher expressions of N-cadherin and p-Smad2 and lower E-cadherin expression in the circ-0001875 overexpression group compared with expression in the control group. These results were also obtained after overexpression of SP1 and were reversed by the TGFβ/Smad pathway inhibitor galunisertib. We further observed the presence of abundant filopodia in the circ-0001875 overexpression group compared with that in the vector group. Together, our data indicated that circ-0001875, via the miR-31-5p/SP1 axis, induces NSCLC cell migration and invasion and promotes EMT through the TGFβ/Smad2 signal pathway. Circ-0001875 contributes to the formation of filopodia, which may participate in EMT process.

One of the most common RNA circularization modes depends on the complementary pairing of intron flanks, and Alu elements play a vital function in this process [44]. When Alu bases undergo complementary pairing, this brings the splice sites into close proximity to back splice to form circRNAs [22]. In our study, we found two highly complementary Alu elements, the upstream AluSq element and the downstream AluSz6 element, located in introns 1 and 5 of *FAM120A* pre-mRNA, promoting circ-0001875 circularization. However, study showed that ADAR1 binds Alu elements to perform adenosine to inosine editing and disengages the structure of double-stranded RNA (dsRNA), to impede RNA circularization [23]. DHX9 also has been reported as a nuclear dsRNA resolvase, it binds to Alu elements to prevent splice sites from being close enough to back splice, thus restraining RNA circularization [38]. Previous reports revealed that SP1 binds with high affinity to Alu elements [24, 25], therefore, we speculated whether the binding between SP1 and Alu element could influence the circularization of circ-0001875. RIP assays confirmed that SP1 specifically and directly bound to AluSq element on the 150–154 sites. In addition, RT-qPCR analysis showed that circ-0001875 circularization was hampered in cells overexpressing SP1, while SP1 depletion promoted circ-0001875 production, with no effects on linear *FAM120A* mRNA level. Together, these results indicated that the circularization of circ-0001875 is negatively regulated by SP1 through an AluSq-dependent feedback loop.

Our results showed that the SP1-AluSq feedback loop reduced circ-0001875 circularization, if it goes on like this, the expression of circ-0001875 should be at low levels in NSCLC cells and tissues. However, we detected the significant high expression of circ-0001875 as the above results. Therefore, we speculated what else may be involved in this process. Hypoxia plays key roles in NSCLC growth and positively regulates gene expression [5]. RT-qPCR showed increased levels of circ-0001875 in hypoxia condition compared with levels in normoxia. Some studies showed that SP1 could collaborate with HIF1α to activate gene expression under hypoxia environment [39, 40]. Therefore, we further explored whether hypoxia increased circ-0001875 expression via the HIF1α-SP1 interaction. CoIP confirmed the interaction between HIF1α and SP1 and RT-qPCR analysis showed that HIF1α knockdown reduced circ-0001875 expression under hypoxia. Furthermore, RIP assay has shown the lower affinity between SP1 and AluSq in hypoxia than in normoxia. Together, these results suggested that HIF1α may compete with the AluSq element for binding to SP1 under hypoxia, resulting in the increase of circ-0001875 formation. Together, our results revealed the SP1-AluSq-dependent feedback loop as an antitumor mechanism that can reduce circ-0001875 circularization to restrain the progression of NSCLC to some extent. However, hypoxia occurs during NSCLC tumor growth, and we found that hypoxia of NSCLC cells induced HIF1α competitive binding to SP1; this may relieve the SP1-Alu feedback suppression loop and increase circ-0001875 circularization. We further found a strong correlation between the expressions of circ-0001875, miR-31-5p and SP1 and NSCLC clinical features and prognosis.

## Conclusion

Here we demonstrate that circ-0001875, identified as a novel circRNA upregulated in lung cancer, regulates the miR-31-5p/SP1 axis with an oncogenic role to promote EMT via a TGFβ/Smad2 signal pathway. The circ-0001875/miR-31-5p/SP1 axis is correlated with tumor clinical features and prognosis of NSCLC patients. Moreover, the regulation axis forms a SP1-AluSq-dependent feedback loop to reduce circ-0001875 circularization, which is disrupted by competitive binding of HIF1α to SP1 under hypoxic condition. This study provided new insights into the regulation mode of circRNA formation under tumor hypoxia.

## Supplementary Information

**Additional file 1: Figure S1.** Circ_0001875 promotes the proliferation, migrationand invasion of NSCLC cells in vitro.

**Additional file 2: Figure S2.** Circ_0001875 knockdown suppressed the tumorigenesisand metastasis of NSCLC cells *in vivo*.

**Additional file 3: Figure S3.** Circ_0001875 acts as a sponge for miR-31-5p.

**Additional file 4: Figure S4.** Silencing ofSP1 reverses the proliferation, migration and invasion induced by circ-0001875overexpression.

**Additional file 5: Table S1.** probes and siRNAs used in the experiments.

**Additional file 6: Table S2.** Primer used in the experiments.

**Additional file 7: Table S3.** Antibodies used in the present study.

**Additional file 8: Table S4.** 21 candidates miRNAs binding to circ-0001875predicted by CircInteractome.

## Data Availability

All data generated or analyzed during this study are included in this published article.

## Abbreviations

circRNA: Circular RNA
miRNA: MicroRNA
NSCLC: Non-small cell lung cancer
EMT: Epithelial-to-mesenchymal transition
TGFβ: Transforming growth factor beta
Smad2: SMAD family member 2
SP1: Specificity protein 1
HIF1α: Hypoxia-inducible factor-1α
qRT-PCR: Quantitative reverse transcription polymerase chain reaction
RIP: RNA immunoprecipitation
CoIP: Co-immunoprecipitation
FISH: Fluorescent in situ hybridization
3′UTR: 3′-Untranslated regions
dsRNA: Double-stranded RNA
DAPI: 4,6-Diamidino-2-phenylindole
CCK-8: Cell count kit-8
siRNA: Short interfering RNA
IHC: Immunohistochemistry
DMEM: Dulbecco’s modified Eagle’s medium
FBS: Fetal calf serum

## Footnotes

1 TNM stage: The staging system of tumors (tumor size-T, node metastasis-N, distant metastasis-M).
