# Supplementary material for: HIF1α-SP1 interaction disrupts the circ-0001875/miR-31-5p/SP1 regulatory loop under a hypoxic microenvironment and promotes non-small cell lung cancer progression
Source: J Exp Clin Cancer Res. 2022 Apr 27;41:156. doi: 10.1186/s13046-022-02336-y (PMC9044860; doi:10.1186/s13046-022-02336-y)
Supplement: Supplementary file 2 — Additional file 2: Figure S2. Circ_0001875 knockdown suppressed the tumorigenesisand metastasis of NSCLC cells in vivo. [file 13046_2022_2336_MOESM2_ESM.docx]

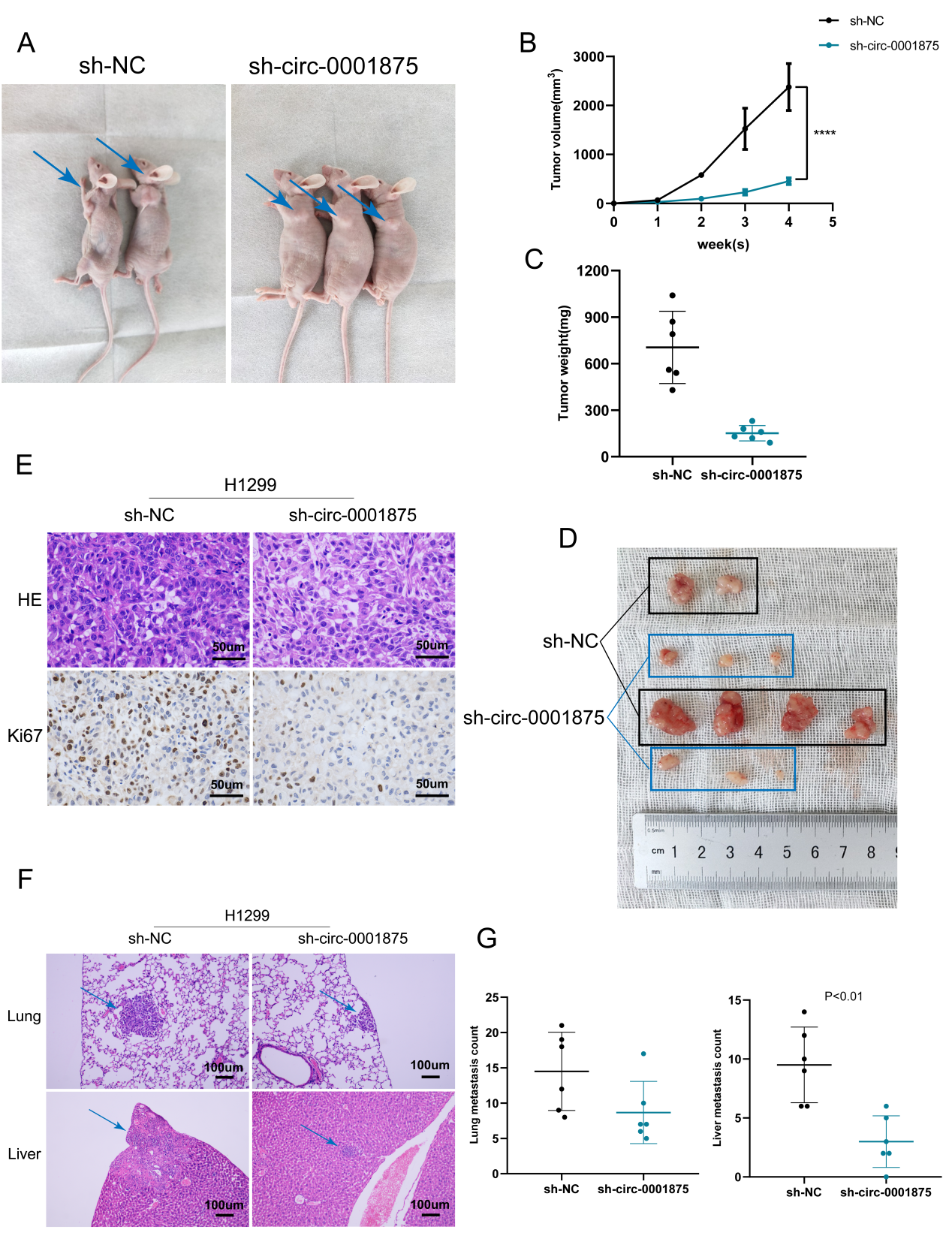


**Fig. S2 Circ_0001875 knockdown suppressed the tumorigenesis and metastasis of NSCLC cells *in vivo*.** **a.** Subcutaneous tumor nude mouse model generated using H1299 cells (n = 6 for each group). **b.** Tumor volume was measured every week. **c–d.** Tumor weight curves and representative images of tumors from both groups. **e.** HE and Ki67 IHC staining of xenograft tumors; images are shown at 400× magnification. Scale bar = 50 μm. **f.** Representative images of HE staining in tail vein–injected mouse models (n = 6 for each group), images are shown at 100× magnification. Scale bar = 100 μm. **g.** The numbers of lung and liver metastatic tumors were counted. Data are shown as the means ± SD. **p* < 0.05, ***p* < 0.01, ****p* < 0.001.
