# Supplementary material for: HIF1α-SP1 interaction disrupts the circ-0001875/miR-31-5p/SP1 regulatory loop under a hypoxic microenvironment and promotes non-small cell lung cancer progression
Source: J Exp Clin Cancer Res. 2022 Apr 27;41:156. doi: 10.1186/s13046-022-02336-y (PMC9044860; doi:10.1186/s13046-022-02336-y)
Supplement: Supplementary file 2 [file 13046_2022_2336_MOESM5_ESM.docx]

Table S1.probes and siRNAs used in the experiments.

Gene	Sequence (5’-3’)
Negative control	sense: UUCUCCGAACGUGUCACGUTT<br>antisense: ACGUGACACGUUCGGAGAATT
si-circ-0001875-1	sense: GUUGGAUUCAUUACACCAC<br>antisense: CAACCUAAGUAAUGUGGUG
si-circ-0001875-2	sense: CACAUUACUUAGGUUGCAC<br>antisense: GUGCAACCUAAGUAAUGUG
miR-31-5p mimics	sense: AGGCAAGAUGCUGGCAUAGCU<br>antisense: CUAUGCCAGCAUCUUGCCUUU
inhibitor NC	CAGUACUUUUGUGUAGUACAA
miR-31-5p inhibitor	AGCUAUGCCAGCAUCUUGCCU
si-SP1-379	sense: CCUCACAGCCACACAACUUTT<br>antisense: AAGUUGUGUGGCUGUGAGGTT
si-SP1-762	sense: GCAACAUCAUUGCUGCUAUTT<br>antisense: AUAGCAGCAAUGAUGUUGCTT
si-SP1-1689	sense: CCAUUAACCUCAGUGCAUUTT<br>antisense: AAUGCACUGAGGUUAAUGGTT
si-HIF-1α	sense: CUCCCUAUAUCCCAAUGGATT<br>antisense: UCCAUUGGGAUAUAGGGAGTT
pcDNA circ-0001875	agtagagacggggtttcaccatgttggccaggctggtcttCACTTTTTGTAAAGGTACGTACTAATGACTTTTTTTTTAT<br>CTTCAGGTTGCACAGAGCATTGAGGATCACCATCAGGAAGTGATTGGTTTCTGCAGAGAGAATG<br>GTTTCCATGGCTTGGTTGCGTATGACTCTGATTATGCACTGTGCAACATCCCCTACTATTTCAGT<br>CCCATGCCCTAAAACTGAGCCGGAACGGGAAAAGTCTCACCACAAGCCAATATCTGATGCATGA<br>AGTTGCCAAGCAACTGGACCTGAATCCAAATCGTTTTCCTATTTTTGCTGCTCTCTTAGGAAATCA<br>CATTCTGCCTGATGAAGATCTGGCTTCCTTTCACTGGAGTTTACTTGGTCCAGAACATCCACTAG<br>CCTCACTAAAGGTCCGGGCCCACCAGCTGGTCTTGCCACCTTGCGACGTAGTGATCAAAGCCG<br>TGCTGACTATGTACGCAACATTCAGGACACCTCTGACTTGGATGCCATAGCTAAAGATGTTTTCC<br>AGCATTCACAGTCTAGAACAGATGACAAAGTTATTCGATTTAAGAGAGCAATTGGATATTATTCAG<br>CGACTAGTAAGCCTATGTCATTTCATCCACCACATTACTTAGGTAAGAAGCAAGGAAAAGAATTA<br>agaccagcctggccaacatggtgaaaccttgtctctact
pcDNA SP1	atgagcgaccaagatcactccatggatgaaatgacagctgtggtgaaaattgaaaaaggagttggtggcaataatgggggcaatggtaatggtg<br>tggtgccttttcacaggctcgaagtagcagcacaggcagtagcagcagcactggaggaggagggcaggagtcccagccatcccctttggctc<br>gctggcagcaacttgcagcagaattgagtcacccaatgagaacagcaacaactcccagggcccgagtcagtcaggggggaacaggtgagct<br>gacctcacagccacacaactttcacagggtgccaatggctggcagatcatctcttcctcctctggggctacccctacctcaaaggaacagagt<br>gcagcagtaccaatggcagcaatggcagtgagtcttccaagaatcgcacagtctctggtgggcagtatgttgtggctgccgctcccaacttaca<br>aaccagcaagttctgacaggactacctggagtgatgcctaatattcagtatcaagtaatcccacagttccagaccgttgatgggcaacagctgc<br>gtttgctgccactggggcccaagtgcagcaggatggttctggtcaaatacagatcataccaggtgcaaaccaacagattatcacaaatcgagg<br>agtggaggcaacatcattgctgctatgccaaacctactccagcaggctgtcccctccaaggcctggctaataatgtactctcaggacagactc<br>gtatgtgaccaatgtaccagtggccctgaatgggaacatcaccttgctacctgtcaacagcgtttctgcagctaccttgactcccagctctcaggc<br>gtcacgatcagcagctctgggtcccaggagagtggctcacagcctgtcacctcagggactaccatcagttctgccagcttggtatcatcacaa<br>cagttccagctcctttttcaccaatgccaatagctactcaactactactaccaccagcaacatgggaattatgaactttactaccagtggatcatca<br>ggaccaactctcaaggccagacaccccagagggtcagtgggctacaggggtctgatgctctgaacatccagcaaaaccagacatctggag<br>ctcattgcaagcaggccagcaaaaagaaggagagcaaaaccagcagacacagcagcaacaaattcttatccagcctcagctagttcaagg<br>ggacaggccctccaggccctccaagcagcaccattgtcagggcagacctttacaactcaagccatctcccaggaaaccctccagaacctc<br>agcttcaggctgttccaaactctggtcccatcatcatccggacaccaacagtggggcccaatggacaggtcagttggcagactctacagctgc<br>gaacctccaagttcagaacccacaagcccaaacaatcaccttagccccaatgcagggtgtttccttggggcagaccagcagcagcaacacc<br>actctcacacccattgcctcagctgcttccattcctgctggcacagtcactgtgaatgctgctcaactctcctccatgccaggcctccagaccatt<br>acctcagtgcattgggtacttcaggaatccaggtgcacccaattcaaggcctgccgttggctatagcaaatgccccaggtgatcatggagctca<br>cttggtctccatggggctggtggtgatggaatacatgatgacacagcaggtggagaggaaggagaaaacagcccagatgcccaaccccaag<br>cggtcggaggacccggcgggaagcatgcacctgcccctactgtaaagacagtgaaggaaggggctcgggggatcctggcaaaaagaaac<br>gcatatttgccacatccaaggctgtgggaaagtgtatggcaagacctctcacctgcgggcacacttgcgctggcatacaggcgagaggccattt<br>tgtgtacctggtcatactgtgggaaacgcttcacacgttcggatgagctacagaggcacaaacgtacacacacaggtgagaagaaatttgcctg<br>cctgagtgtcctaagcgcttcatgaggagtgaccacctgtcaaaacatatcaagacccaccagaataagaagggaggcccaggtgtagctct<br>agtgtgggcactttgcccctggacagtggggcaggttcagaaggcagtggcactgccactccttcagcccttattaccaccaatatggtagccat<br>gaggccatctgtccagagggcattgcccgtcttgccaacagtggcatcaacgtcatgcaggtggcagatctgcagtccattaatatcagtggca<br>ggcttctga
