# Supplementary material for: HIF1α-SP1 interaction disrupts the circ-0001875/miR-31-5p/SP1 regulatory loop under a hypoxic microenvironment and promotes non-small cell lung cancer progression
Source: J Exp Clin Cancer Res. 2022 Apr 27;41:156. doi: 10.1186/s13046-022-02336-y (PMC9044860; doi:10.1186/s13046-022-02336-y)
Supplement: Supplementary file 3 [file 13046_2022_2336_MOESM6_ESM.docx]

Table S2. Primer used in the experiments.

Gene	Sequence (5'-3')	Application
Hsa_circ:chr3:169854206-169867032	F: CAAGCCTGGAATCACGAAGC<br>R: GTTGGGTAATACTGCCGCTG	qRT-PCR
Hsa_circ:chr9:138709822-138774924	F: AAGTCAAAGCCGAAGAAGCC<br>R: CCTGCTCATACTGGTCAACG	qRT-PCR
Hsa_circ:chr4:187560875-187584767	F: ACGTCAAGGAAGCTAGACCG<br>R: TGAACGATGAAAGAGGCACG	qRT-PCR
Hsa_circ:chr9:96277948-96238551	F: ACGTGGATTCCGCCTACTTC<br>R: AGAGGTCTGCGGAATGTTGA	qRT-PCR
Hsa_circ:chr20:30954167-30956926	F: CCTCTCGCATGCCTCAATG<br>R: TGTTTTGGTGTCATTGGAGCA	qRT-PCR
Hsa_circ:chr5:138614015-138651427	F: GGTTATCCCCATCTGTGCTCT<br>R: ACCTGCCACTATTTCCTCCC	qRT-PCR
Hsa_circ:chr9:102677459-102691125	F: TCTGCTCGTCTTCCAACATC<br>R: CCTTGACACAGGTGCCAT	qRT-PCR
Hsa_circ_0001875	F: ACCACATTACTTAGGTTGCACAG<br>R: CGTTCCGGCTCAGTTTTAGG	qRT-PCR
hsa_circ_0001875(convergent)	F: TACGCAACATTCAGGACACC<br>R: GAGTCATACGCAACCAAGCC	qRT-PCR
β-actin	F: TCTCCCAAGTCCACACAGG<br>R: GGCACGAAGGCTCATCA	qRT-PCR
U6	F: CTCGCTTCGGCAGCACA	qRT-PCR
hsa-miR-31-5p	F: AGGCAAGATGCTGGCATAGCT	qRT-PCR
SP1	F: TTTTGATGTGTGGGCTTCT<br>R: TGGCTGATGCTCCTTATTG	qRT-PCR
HIF-1α	F: GCAGCAACGACACAGAAA<br>R: AGCGGTGGGTAATGGAG	qRT-PCR
AluSq WT	F: TAGCTGGGATTATAGGCGCCCGCC<br>R: TCACACCTGTAATCCCAGCA	qRT-PCR
AluSq MUT (150-154)	F: AGCTGGGATTATAGGCGCAATAA<br>R: TCACACCTGTAATCCCAGCA	qRT-PCR
