# Supplementary material for: HIF1α-SP1 interaction disrupts the circ-0001875/miR-31-5p/SP1 regulatory loop under a hypoxic microenvironment and promotes non-small cell lung cancer progression
Source: J Exp Clin Cancer Res. 2022 Apr 27;41:156. doi: 10.1186/s13046-022-02336-y (PMC9044860; doi:10.1186/s13046-022-02336-y)
Supplement: Supplementary file 4 [file 13046_2022_2336_MOESM7_ESM.docx]

Table S3. Antibodies used in the present study.

Product	Source	No. of Catalogue
**Western blot:**		
anti-β-actin	GeneTex	GTX109639
anti-E-cadherin	Proteintech	20874-1-AP
anti-N-cadherin	Proteintech	22018-1-AP
anti-SP1	Cell Signaling Technology	9389S
anti-Phospho-smad2 (Ser465/Ser467)	Cell Signaling Technology	18338S
anti-Smad2	Cell Signaling Technology	5339S
anti-HIF-1α	abcam	ab51608
**Secondary antibody**		
anti-rabbit IgG-HRP	Proteintech	SA00001-15
**IHC:**		
Anti-Ki67	Abcam	ab15580
anti-SP1	Cell Signaling Technology	9389S
**Secondary antibody**		
anti-rabbit IgG-HRP	Proteintech	SA00001-15
**RIP:**		
IgG	Merck	PP64B
anti-SP1	Cell Signaling Technology	9389S
**CoIP:**		
IgG	Cell Signaling Technology	3900S
anti-SP1	Cell Signaling Technology	9389S
anti-HIF-1α	abcam	ab51608
