# Supplementary material for: HIF1α-SP1 interaction disrupts the circ-0001875/miR-31-5p/SP1 regulatory loop under a hypoxic microenvironment and promotes non-small cell lung cancer progression
Source: J Exp Clin Cancer Res. 2022 Apr 27;41:156. doi: 10.1186/s13046-022-02336-y (PMC9044860; doi:10.1186/s13046-022-02336-y)
Supplement: Supplementary file 5 [file 13046_2022_2336_MOESM8_ESM.docx]

Table S4. 21 candidate miRNAs binding to circ-0001875 predicted by CircInteractome.

Seq	miRNA
hsa_circ:chr9:96233422–96261168	hsa-miR-151a
hsa_circ:chr9:96233422–96261168	hsa-miR-1287
hsa_circ:chr9:96233422–96261168	hsa-miR-891b
hsa_circ:chr9:96233422–96261168	hsa-miR-203a
hsa_circ:chr9:96233422–96261168	hsa-miR-516b
hsa_circ:chr9:96233422–96261168	hsa-miR-409-3p
hsa_circ:chr9:96233422–96261168	hsa-miR-384
hsa_circ:chr9:96233422–96261168	hsa-miR-1825
hsa_circ:chr9:96233422–96261168	hsa-miR-182
hsa_circ:chr9:96233422–96261168	hsa-miR-183
hsa_circ:chr9:96233422–96261168	hsa-miR-296-5p
hsa_circ:chr9:96233422–96261168	hsa-miR-145
hsa_circ:chr9:96233422–96261168	hsa-miR-31-5p
hsa_circ:chr9:96233422–96261168	hsa-miR-1200
hsa_circ:chr9:96233422–96261168	hsa-miR-1184
hsa_circ:chr9:96233422–96261168	hsa-miR-1299
hsa_circ:chr9:96233422–96261168	hsa-miR-1257
hsa_circ:chr9:96233422–96261168	hsa-miR-1205
hsa_circ:chr9:96233422–96261168	hsa-miR-921
hsa_circ:chr9:96233422–96261168	hsa-miR-545
hsa_circ:chr9:96233422–96261168	hsa-miR-548p
